# 20 Years of Trans Visibility, from Mourning to Fighting!

**DOI:** 10.1590/S2237-96222024V33E2024034.ESPECIAL.EN

**Published:** 2024-01-26

**Authors:** Keila Simpson, Bruna Benevides

**Affiliations:** 1 Associação Nacional de Travestis e Transexuais (ANTRA), Salvador, Bahia, Brazil; 2 Marinha do Brasil, Niterói, Rio de Janeiro, Brazil

Twenty years later, we travestis are here to continue marking the important changes that have been taking place, since a group organized within the Associação Nacional de Travestis e Transexuais - ANTRA, entered, for the first time, the National Congress, to launch the Campaign “Travesti e Respeito”, together with representatives of the then National STD/AIDS Program, part of the Brazilian Health Ministry’s Health Surveillance Secretariat, which today bears the name of Department of HIV/AIDS, Tuberculosis, Viral Hepatitis and Sexually Transmitted Infections, within the Health Ministry’s Health and Environment Surveillance Secretariat (Departamento de HIV/Aids, Tuberculose, Hepatites Virais e Infecções Sexualmente Transmissíveis da Secretaria de Vigilância em Saúde e Ambiente do Ministério da Saúde - DATHI/SVSA/MS). In this way, ANTRA conceived the creation of National Trans Visibility Day in Brazil, remembering a historical date that began there, with the aim that this date would be celebrated in the years that followed.

Established in 2004, national Trans Visibility emerged five years before International Trans Visibility Day,^1^ celebrated since March 31, 2009, which demonstrates our precedence in relation to the movement of *travestis* politically mobilized around the world.

“Travesti and respect: it’s time for both of them to be seen alongside each other. At home. At the nightclub. At school. At work. In life”. The Travesti and Respect Campaign was launched at the National Congress, on January 29, 2004, and is focused on reinforcing attitudes of respect and social inclusion for this segment of the population, which is very vulnerable to the AIDS virus due to prejudice and violence. The campaign was carried out by leaders of the organized movement of travestis and transsexuals linked to ANTRA, in partnership with the National STD/Aids Program, and has four targets to reach: schools, health services, the community and clients of *travestis* sex workers. The slogan is reproduced on posters and folders with photos of the 27 *travestis* who participated in the creation of the campaign.^2^

Years have gone by and trans visibility has increasingly mobilized efforts to take steps forward, promoting structural changes in order to guarantee access to rights and citizenship for travestis and transsexual people. And in this process of changing the political scenario and the possibility of reclaiming their voices, *travestis* continue to act to affirm that, although initially they started from a situation of complete absence of rights and obstacles to a dignified existence, today they remain undeterred, envisioning the dreams that at that time were so far from being transformed into the reality that has resulted from what they have fought for.

Many *travestis* survived the police operations that persecuted them during the dictatorship^3^ and the HIV/AIDS epidemic that decimated a large number of fellow fighters and which still persists in the Brazilian trans reality.^4^ Albeit contradictory, this epidemic also enabled the building of the capacity of many *travestis* on this issue, first as community health educators, and then coordinating projects and even developing research related to this condition. Furthermore, it was important for the political awareness of many of them, since many have considered themselves to be the *daughters of aids*. Survivors of epidemics of hatred against gender identities,^5^ they have transformed Brazil and helped to rebuild the nation and its young democracy, promoting debate on the urgency of respecting bodily, sexual and gender diversity, in a qualified and transformative manner, for the whole of society.

This debate, like so many others, also concerns the visibility that trans and travestis people have gained, especially in recent years, so that today they no longer want or accept living within invisibility, confined to ghettos, or limiting themselves to going out only at night, as occurred in the past. Unfortunately, this altruistic conquest of spaces that *travestis* claim (even in the adverse scenario in which they live) is still mixed with the violence suffered by its victims when claiming their freedom to be who they are.

It is when *travestis* start to live in wider social spaces and have access to rights previously denied, that they really cause discomfort. They quickly began to understand this issue, and with every day that passes they are becoming increasingly aware of their social conditions - achieving a certain degree of citizenship, albeit precarious - and of the rights guaranteed to them by the struggle undertaken by people-based social movements. Furthermore, they experience the possibility of building and coexisting with other forms of affective/sexual relationships, as well as access to work spaces that were previously prohibited.

This autonomy achieved through the guarantees provided by rights they have conquered has created the idea that there are new perspectives for the future, and that one can think beyond prostitution - if that is one’s personal desire. It was the struggle of the “birth-giving *travestis*” that conceived and gave birth to other work fronts for the trans population, whereby a small degree of progress in the autonomy of these people has been seen. However, within the scenario of labor and social security reform, brought about by the resurgence of neoliberal policies introduced by the two previous governments, this progress dissipated. This led to several harmful impacts on the trans community, and many *travestis* returned to sex work.

It is well known that Brazil is a country of continental dimensions, and public policies are not balanced or available in the same proportion for all people. However, having a legal system the highest level of which is, at this moment, committed to correcting injustices and omissions brings hope to those who remain invisible, in their social coexistence and even in the development of their professional skills. It should be noted that there remains a huge shortfall with regard to the issue of work, which needs to be corrected. Obviously, initiatives by social organizations in this field are welcome and important, but it is necessary to have government employment and income generation mechanisms and policies, monitored through social watchdog bodies, that actually address this issue. It should be noted that since there is a population of young trans people who are experiencing a new moment, having access to education and training, albeit insufficiently, it is necessary to move forward, since the violence they suffer in the education system is still of considerable concern. Similarly, it will be necessary to prepare the job market, institutions and society for this new cycle of professionals.

If, on the one hand, the trans movement has achieved a great deal of progress along this journey - which places Brazil as one of the most advanced countries in achieving these rights -,^6^ on the other hand there are still challenges to transforming them into public policies and ensuring access for all trans and travestis people, breaking with the paradox of violence and promoting the recognition of these people’s citizenship.

To this end, we need, for example, to commit ourselves to interrupting the transphobic ideological debate, which today has elected transphobia as a priority political agenda of the far right^7^ and other anti-trans groups that have mobilized themselves around an anti-gender agenda, creating caricatured and ghostly allegories in order to fuel anti-trans panic.

In this scenario, it is imperative to affirm that the violation of rights and violence, which remain naturalized against the trans population, should no longer go unchallenged. Murders, still very present in this segment, cannot discourage the continued active search for a solution to eradicate them. The incessant demand for more urgent and comprehensive social inclusion has become one of the most important battles flags today and, together with the continuation of institutional activities - establishing important partnerships for this fight -, it will always be a day-to-day task, demanding spaces for inclusion.

We have been molded by our fight and we will not deter from it until our rights are guaranteed. We want rights in full and not by halves. We need legislation that guarantees the prevention of violence, investigative measures - with criminals held accountable - and collective reparation, as we will not hesitate to combat omission and impunity. This action is plural, and only collective action makes sense. We must always keep at bay violent and transphobic discourses, and the statements of fundamentalists and gender essentialists, ultra-conservative anti-rights groups, racists, sexists, misogynists, trans-exclusionary groups and any attempt whatsoever aimed at persecution, silencing or violence must be loudly repudiated.

In this sense, this collection of articles, in addition to marking 20 years of Trans Visibility, calls on all trans people, *travestis* and allies, individuals and institutions, to engage decisively in the fight to defend the human rights of this part of the population. This fight is not just about a single person or movement, and we hope to make many more achievements on this journey over the forthcoming years.

We believe in the reestablishment of democracy and social inclusion, and we invite anyone willing to walk by our side at this time when trans people are on the front line of this fight!
